# Ecological Complexity of Coral Recruitment Processes: Effects of Invertebrate Herbivores on Coral Recruitment and Growth Depends Upon Substratum Properties and Coral Species

**DOI:** 10.1371/journal.pone.0072830

**Published:** 2013-09-09

**Authors:** Sarah W. Davies, Mikhail V. Matz, Peter D. Vize

**Affiliations:** 1 Integrative Biology Section, The University of Texas at Austin, Austin, Texas, United States of America; 2 Department of Biological Sciences, University of Calgary, Calgary, Alberta, Canada; University of Otago, New Zealand

## Abstract

**Background:**

The transition from planktonic planula to sessile adult corals occurs at low frequencies and post settlement mortality is extremely high. Herbivores promote settlement by reducing algal competition. This study investigates whether invertebrate herbivory might be modulated by other ecological factors such as substrata variations and coral species identity.

**Methodology/Principal Findings:**

The experiment was conducted at the Flower Garden Banks, one of the few Atlantic reefs not experiencing considerable degradation. Tiles of differing texture and orientation were kept in bins surrounded by reef (24 m). Controls contained no herbivores while treatment bins contained urchins (*Diadema antillarum*) or herbivorous gastropods (*Cerithium litteratum*). Juvenile corals settling naturally were monitored by photography for 14 months to evaluate the effects of invertebrate herbivory and substratum properties. Herbivory reduced algae cover in urchin treatments. Two genera of brooding coral juveniles were observed, *Agaricia* and *Porites*, both of which are common but not dominant on adjacent reef. No broadcast spawning corals were observed on tiles. Overall, juveniles were more abundant in urchin treatments and on vertical, rough textured surfaces. Although more abundant, *Agaricia* juveniles were smaller in urchin treatments, presumably due to destructive overgrazing. Still, *Agaricia* growth increased with herbivory and substrata texture-orientation interactions were observed with reduced growth on rough tiles in control treatments and increased growth on vertical tiles in herbivore treatments. In contrast to *Agaricia, Porites* juveniles were larger on horizontal tiles, irrespective of herbivore treatment. Mortality was affected by substrata orientation with vertical surfaces increasing coral survival.

**Conclusions/Significance:**

We further substantiate that invertebrate herbivores play major roles in early settlement processes of corals and highlight the need for deeper understanding of ecological interactions modulating these effects. The absence of broadcast-spawning corals, even on a reef with consistently high coral cover, continues to expose the recruitment failure of these reef-building corals throughout the Caribbean.

## Introduction

Coral reefs are globally threatened and many reefs are experiencing ecological shifts to algae-dominated communities [1]–[3], especially in the Caribbean [4], [5]. Competition for space and overgrowth by algae appear to be major factors affecting coral recruitment and survival [3], [6]–[10]. Algae are effective competitors for space, especially after large-scale disturbances [11] and once algae are established, coral recruitment can be suppressed [3], [5], [12]. Herbivore exclusion experiments have shown that grazer removal increases both algal growth and coral mortality [8], [9], [13], [14], while herbivore inclusion generally enhances coral recruitment [15]–[17].

Following severe reef fish exploitation in the early 20^th^ century, herbivory in the Caribbean became dominated by an invertebrate grazer, the long-spined sea urchin (*Diadema antillarum*) [1], [18], [19]. In the 1970’s *D. antillarum* densities were generally high [20], [21] and were recorded as great as 71 m^−1^ [22]; however, in 1982, a species-specific pathogen induced *D. antillarum* mortality across the Caribbean reaching 99% in some localities [1], [21], [23]. Soon after this mortality event, coral recruitment decreased and algal biomass increased [1], [24]. Between 1977 and 1993, Hughes [1] reported an increase in algal cover from 4% to 92% and a drop in coral cover from 52% to 3% on Caribbean reefs. *D. antillarum* recovery has been slow but several Caribbean regions have experienced sea urchin resurgence, which has been correlated with recent enhanced coral recruitment [16], indicating that this invertebrate herbivore may play an important role in reef recovery.

Although most Caribbean reefs are in a state of decline, one of the few exceptions is the Flower Garden Banks (FGB) National Marine Sanctuary in the Gulf of Mexico. In contrast to many other Caribbean reefs, coral cover at the FGB has been consistently high over the past decades [25], [26] and fish populations are robust with herbivorous fishes densities of 4.43 fish per 100 m^2^ [27]. The banks are also unique since they comprise the northernmost coral reef in the continental United States and are relatively deep when compared to other Caribbean reefs (>20 m). The coral cover and location of these reefs make them an ideal location for investigating processes affecting coral settlement and survival.

Coral recruitment has been shown to be quite complex and this particular life-history stage has received much attention (e.g. [28]). Areas of recruitment research range from competency variation [29]–[31], to gene expression responses to settlement induction [32]–[34], to the factors affecting recruitment (e.g. [10], [35]). One area of specific interest has been the direct and indirect effect of algae on corals. Studies have demonstrated the negative effects of certain types of algae on coral recruitment (reviewed in [3]) and others have shown the indirect positive recruitment effects of algae removal by herbivore grazing [7], [36]. Research has also identified microhabitat recruitment preferences that vary between environments and coral species [37]–[41], however, to the best of our knowledge, no study to date has explicitly addressed the interactive settlement and survival patterns of corals in response to herbivore grazing and substrata variations. While it is generally assumed that herbivores are beneficial for coral settlement and survival, these benefits may not be equal across coral species and may vary due to local ecology, leading to the possibility of coral community composition, as well as overall reef structure, being modulated by herbivores.

The objectives of this study were to determine how invertebrate herbivores and substratum variations interact to influence coral settlement, mortality and juvenile growth *in situ*. Specifically, we studied the effects of two local invertebrate herbivores (*Diadema antillarum* and *Cerithium litteratum*) on coral genera on an isolated recruitment platform within the East Flower Garden Banks coral reef. Here coral abundance, size, mortality and growth rate were measured on varying experimental substrata and herbivore treatments.

## Methods

### I. Study Site: Flower Garden Banks National Marine Sanctuary (FGBNMS)

**Figure 1:**
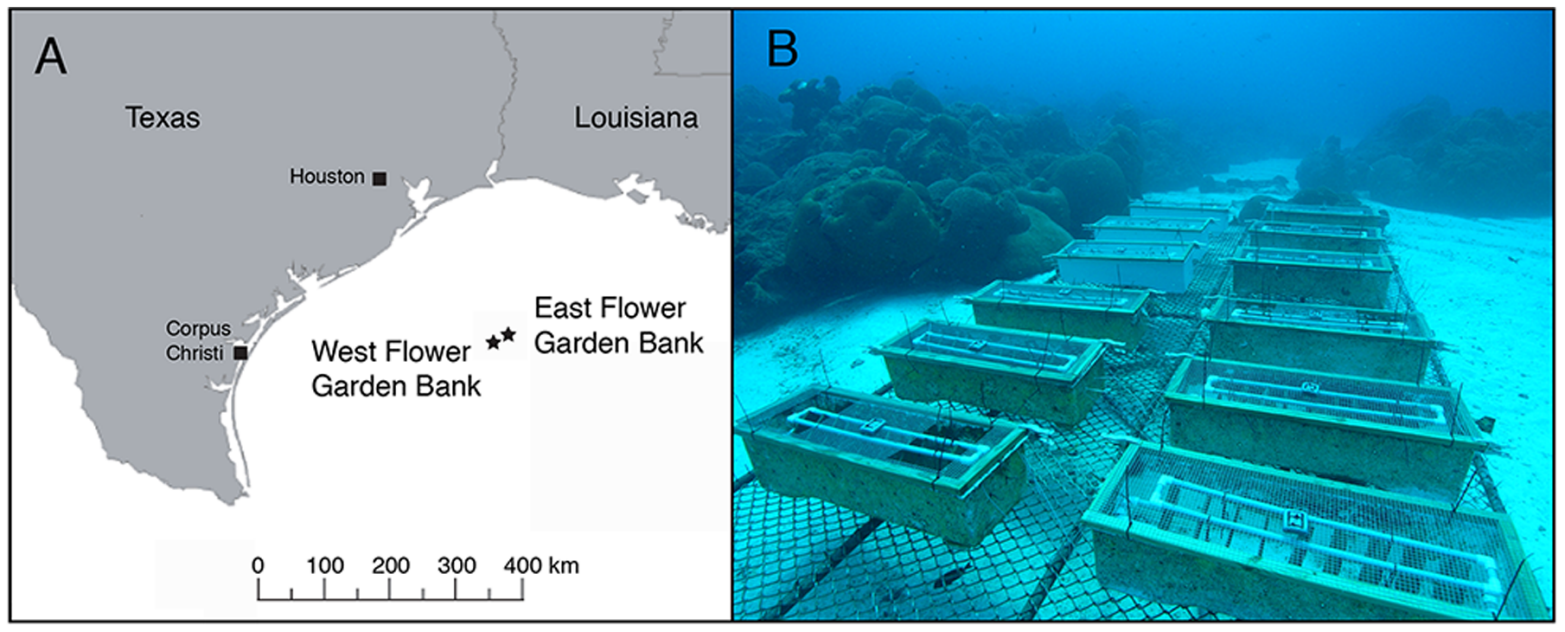
Experimental location and setup. A. Map of the Gulf of Mexico showing the locations of the east and west banks of the Flower Garden Banks National Marine Sanctuary (FGBNMS) (27.92° N; 93.71° W) in relation to continental United States. Figure Credit: USGS. B. Coral recruitment platform established at the east Flower Garden Banks at 24 m depth photographed in August 2008 prior to Hurricane Ike.

The FGBNMS is located 185 km south of the Texas-Louisiana border in the Gulf of Mexico and is a 145.58 km^2^ marine protected area that was designated in January 1992 (Figure 1) [26]. The nearest coral reefs are hundreds of kilometers away along the coast of Tampico, Mexico (645 km) and the Yucatan peninsula (600 km) [42]. Despite this isolation, FGBNMS has been populated with 21 zooxanthellate scleractinian coral species [26]. Water temperatures range from 20–30°C annually, occasionally falling below 18°C, representing the thermal minima for reef building corals. The FGB is one of the few Gulf of Mexico/Caribbean reefs that is not currently experiencing large-scale degradation due to anthropogenic pressures [26], making it particularly suitable for studying Caribbean coral settlement and survival since adult coral density is still very high (56%) [27]. All fieldwork was conducted under permit number FGBNMS-2007–006 issued by the Flower Garden Banks National Marine Sanctuary (FGBNMS) to the University of Calgary, Canada (permitted to Vize).

### II. Experimental Construction and Preparation

A platform measuring 6 m×6 m×0.6 m was constructed at 24 m depth on a sand patch on the east FGB (27°58′28.63′′ N, 93°37′46.67′′ W), Gulf of Mexico in June 2007, three months prior to the annual coral broadcast spawning event (Figure 1). Galvanized steel chain link fence was then attached and served as the top of the platform for experimental equipment support. The platform was anchored to nearby reef using 2 cm galvanized steel chain. Nine fiberglass bins (117 cm×36 cm×25 cm) were attached to the platform using 0.5 cm plastic covered steel cables with aluminum crimps. 0.6 cm holes were drilled on the sides and bottoms of bins to allow for water circulation and sand release. Unglazed, alphagres Spanish red 15 cm×15 cm quarry tiles were seasoned by submersion in sand for several days prior to installation. Twelve tiles were attached to PVC suspension racks and installed in each bin to serve as settlement substrata. Tiles had one smooth and one rough textured side and were placed in two orientations (vertical 90°, horizontal 180°) on PVC suspension racks. Textured tiles had nine parallel indentations (1 mm deep) that were 2 mm in width each followed by one wide indentation (1 mm deep) that was 1.2 cm wide. This pattern repeated N = 5 times across the tile.

Bins were randomly assigned to one of the three experimental herbivore treatments (N = 3): (1) sea urchin (*D. antillarum*) stocked at two individuals per bin, (2) cerith snail (*C. litteratum*) stocked at 50 individuals per bin, and (3) no herbivore control. *D. antillarum* densities were similar to those previously studied by Sammarco [14]. To prevent escape or predation of herbivores, all bins were covered with 1.3 cm^2^ wire mesh.

### III. Data Collection

Figure S1 depicts the experimental timeline. After three months, just prior to coral spawning (September 2007), four tiles from each bin were photographed underwater using a Canon Powershot A75 Digital 3.2 Megapixel camera to assess initial tile community composition. Tiles were returned and left in bins over the winter, but storms caused significant platform damage and sand deposition, so three of the bins (one per herbivore treatment) were removed from all subsequent analyses. After ten months (April 2008), all tiles from treatment bins were brought aboard ship and photographed using a 10.0 Mega-pixel Sea and Sea DX1G digital camera. Tiles were kept immersed at all times except for when images were captured. Tiles were returned to their experimental bins for four additional months, at which point (August 2008) all tiles were permanently removed for the final size, growth and mortality analyses.

### IV. Image Analysis

All images were analyzed using Photoshop 7.0 and ImageJ 10.2. Depending on quality, some images were color and contrast enhanced. To determine percent algal cover on tiles after 3 months, the Photoshop magic wand tool was used to highlight all pixels containing algae and these pixels were deleted. Images were converted to 8-bit in ImageJ and thresholds were adjusted to 0∶254. The “Analyze particles” command calculated area in pixels^2^ and this value was converted to the algae percent cover per tile. Only algae cover was analyzed from the 3-month time point since coral recruits were either not present or too small to be accurately identified given the image resolution. Algae cover from ten and fourteen month time points was not analyzed because recruitment of encrusting species on tiles made differentiating algae from other organisms impossible. Since coral juveniles are radially symmetrical, they were easily identifiable on tiles; however we cannot rule out the possibility that some corals evaded detection due to overgrowth or fouling by other encrusting organisms.

From ten and fourteen month photographs, all corals, regardless of species and size, were counted to obtain the total number of corals per tile. Coral size was determined as its image area, in mm^2^. Coral growth (increase in area) over the four summer months was determined by monitoring individuals that were identified in both sampling periods. These growth data are only presented for *Agaricia* since the sample size for *Porites* was too small. Mortality between sampling points was also quantified.

### V. Statistical Analyses

All statistical analyses were implemented using R software [43] using linear modeling approach (function *lm()*). For all analyses, three fixed factors were studied: experimental bin was nested within herbivore treatment, with levels of sea urchins, snails and no herbivore control, tile texture with levels of smooth and rough, and orientation with levels of horizontal and vertical. A series of nested models omitting one, two, or all three of these fixed factors was fitted in each case and compared to estimate the significance of factors based on the likelihood ratio test (LRT). If factors were found to be significant, post-hoc Tukey's HSD tests were used to evaluate the significance of each pair-wise comparison. All of the assumptions of parametric testing were validated using diagnostic plots in R. The data for plotting were produced using the function *summarySE()* and plotted using package *ggplot2* as described in http://www.cookbook-r.com/Graphs/Plotting_means_and_error_bars_(ggplot2)/. All R scripts and data are provided as electronic supplementary information.

To examine the effects of herbivore treatment, tile texture and tile orientation on the proportion of algae after three months, algal cover data were arcsine square root transformed and LRT and Tukey's HSD tests were performed. LRT was also used to determine if the numbers of corals per tile (at ten and fourteen months) and the arcsine squareroot-transformed mortality proportions were affected by the same covariates as outlined above. Corals were then split by genus and LRTs were run on log-transformed coral size and log-transformed coral growth data. To determine if mortality or growth were correlated with coral density, regressions were run on the number of corals per tile (coral density) against log-transformed coral growth and arcsine squareroot-transformed mortality data.

## Results

### I. Algal Cover at Three Months

LRTs determined that percent algal cover was only significantly affected by three months of herbivore treatment (Table 1, *P_LRT_*  = 0.003). Mean algal cover in bins without herbivores (20.1±3.6%) was significantly higher than cover in the *D. antillarum* treatment (12.2±3.6%) indicating that sea urchin presence reduced algal cover (Tukey's HSD, p = 0.002). Snail presence and tile texture and orientation had no effect on algal cover (Figure S2, Table S1).

**Table 1:** Likelihood ratio test (LRT) statistics for each experiment (algae cover, number of coral juveniles, coral size, coral mortality and *Agaricia* growth) in response to herbivore treatment and tile texture and orientation.

Experiment	Factor	d*f*	SS	F	*p*
Algae Cover	Treatment	2	0.135	6.704	0.003
(3 months)	Residuals	36	0.362		
Coral Numbers	Orientation	1	580.03	58.52	<0.001
(10 months)	Texture	1	256.19	25.85	<0.001
	Orientation*Texture	1	63.88	6.45	0.012
	Residuals	117	1159.7		
Coral Numbers	Treatment	2	860.44	20.06	<0.001
(14 months)	Orientation	1	2609.00	121.64	<0.001
	Texture	1	1392.73	64.94	<0.001
	Orientation*Treatment	2	365.60	8.52	<0.001
	Orientation*Texture	1	372.90	17.39	<0.001
	Orientation*Texture *Treatment	2	152.96	3.57	0.031
	Residuals	114	2445.07		
*Agaricia* Colony	Treatment	2	42.43	22.69	<0.001
Size (10 months)	Orientation	1	82.46	88.17	<0.001
	Texture	1	4.78	5.10	0.024
	Residuals	465	434.85		
*Agaricia* Colony	Treatment	2	119.63	32.93	<0.001
Size (14 months)	Orientation*Texture *Treatment	2	23.60	6.50	0.002
	Residuals	1020	1852.58		
*Porites* Colony	Treatment	2	2.51	3.44	0.038
Size (10 months)	Orientation	1	8.00	21.86	<0.001
	Residuals	71	25.97		
*Porites* Colony	Orientation	1	29.80	34.55	<0.001
Size (14 months)	Residuals	71	61.44		
*Agaricia* Colony	Treatment	2	6.764	8.514	<0.001
Growth	Treatment*Texture	2	3.166	3.985	0.019
	Treatment*Orientation	2	3.291	4.142	0.017
	Residuals	363	144.21		
Coral Juvenile	Orientation	1	0.58	5.04	0.039
Mortality	Residuals	16	1.85		

Only significant terms are included in the table.

### II. Coral Settlement Rates

**Figure 2:**
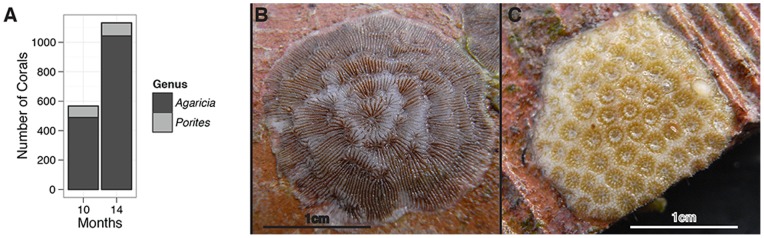
Total number of corals through time. A. Number of corals observed on experimental tiles split by genus 10 and 14 months after deployment at the Flower Garden Banks. B. Photograph of *Agaricia* juvenile on experimental substratum. C. Photograph of *Porites* juvenile on experimental substratum.

Ten and fourteen months after tile deployment, only two coral genera were recorded among juveniles, *Agaricia* and *Porites*. 567 corals were observed on tiles after ten months, 489 of which were *Agaricia* and 78 *Porites.* After fourteen months, 1132 individuals were present, 1043 of which were *Agaricia* and 89 *Porites* (Figure 2). Over the sampling period, *Agaricia* went from being six to eleven times more abundant than *Porites*. The size ranges of the corals observed were 0.44 mm^2^ to 347.9 mm^2^ for *Agaricia* and 0.97 mm^2^ to 156.0 mm^2^ for *Porites.*

**Figure 3:**
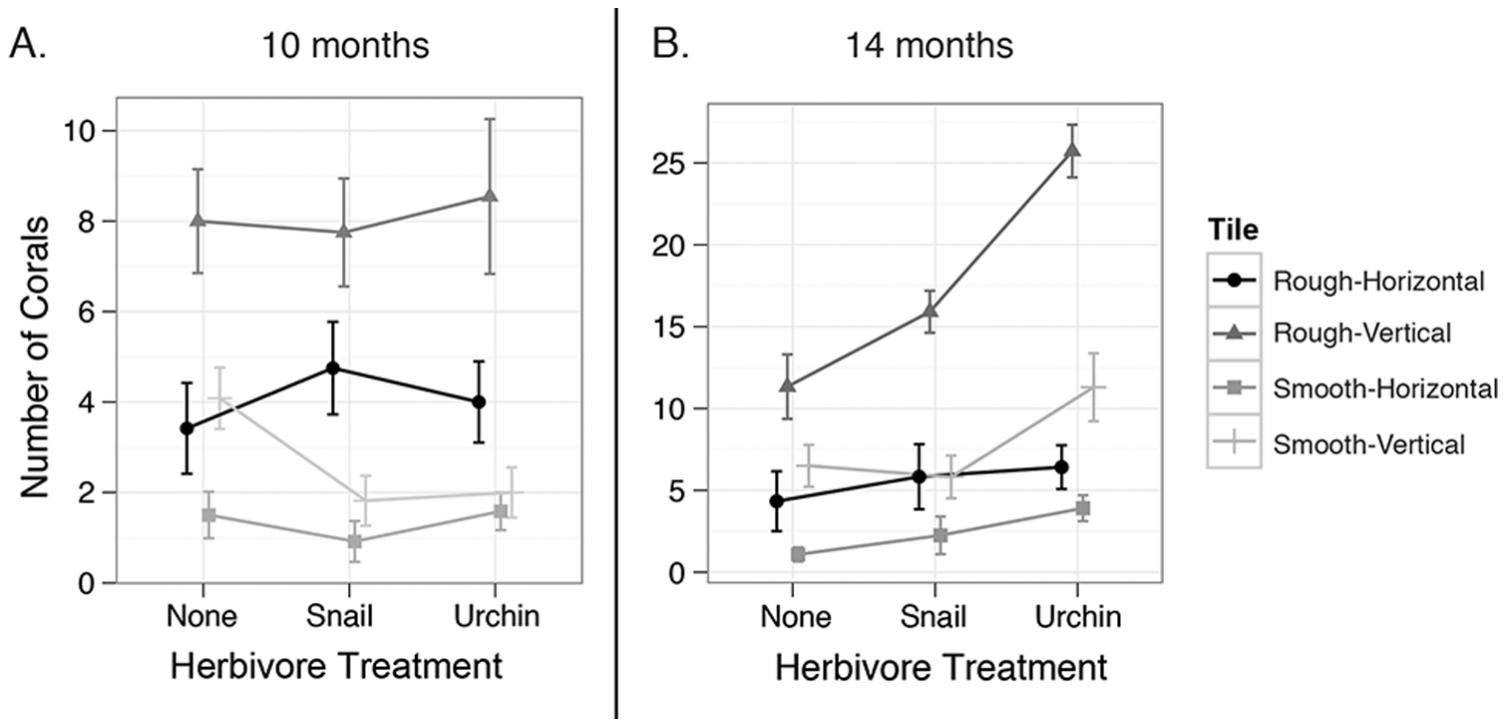
Effects of herbivore and substrata interactions through time. Number of corals per tile depending on herbivore treatment, tile texture and tile orientation at ten (A) and fourteen (B) months. Symbols are means and the whiskers denote 95% confidence intervals.

Herbivore treatment had no effect on the total number of corals on tiles, regardless of species, after ten months; however, tile orientation and texture, as well as their interaction did affect settlement. At ten months, total coral numbers on vertically oriented substrata was twice that of horizontal tiles (5.4±0.5 corals per tile versus 2.7±0.4 corals per tile, Figure 3A, *P_LRT_* <0.0001). At ten months, three-fold more corals were found on rough textured tiles relative to smooth tiles (6.0±0.5 corals per tile versus 2.0±0.2 corals per tile, Figure 3A, *P_LRT_* <0.0001). Significant interactions between texture and orientation were also detected, most likely driven by the overall higher settlement to rough and vertical tiles, although variation in post-settlement mortality across tiles cannot be ruled out.

At fourteen months, the presence of sea urchins had a significant effect on total number of corals (*Agaricia* and *Porites*), resulting in 1.7-fold more corals than the control (*P_LRT_* <0.001), and 1.3-fold more than the snail treatment. At this time point urchin treatments averaged 9.8±1.4 corals per tile, snail treatments 7.3±1.1 corals per tile and control bins 5.8±0.8 corals per tile (Figure 3B). While the snail treatment tiles did have a higher number of corals than control tiles, Tukey's HSD test found no significant difference (Table S1, p = 0.188). Coral recruitment was again more than twice higher on textured tiles (11.3±1.1 corals per tile) than on smooth tiles (5.0±0.6 corals per tile) and three times higher on vertical tiles (12.7±1.0 corals per tile) than horizontal tiles (4.0±0.6 corals per tile) (Figure 3B, *P_LRT_* <0.001). Tukey's HSD tests detected multiple significant differences between interaction terms, the most obvious trends being strong settlement increases in urchin treatments on vertical tiles regardless of texture and increased settlement on rough, horizontal tiles regardless of herbivore treatment (Table S1; Figure 3B).

In summary, corals, regardless of the genus, consistently preferred vertically oriented, rough textured tiles. Herbivore treatment affected algal cover at three months, however it has no effect on coral recruitment at 10 months. Conversely, after 14 months, urchin presence was associated with higher settlement rates. Settlement results were based on data pooled for both *Agaricia* and *Porites* recruits, but it is interesting to note that at ten months *Porites* were over four times more abundant on horizontal tiles (63) than vertically oriented tiles (15), while *Agaricia* followed settlement trends described above. However, both species settled on vertical tiles at higher rates during summer months (14 month time point).

### III. Coral Mortality

**Figure 4:**
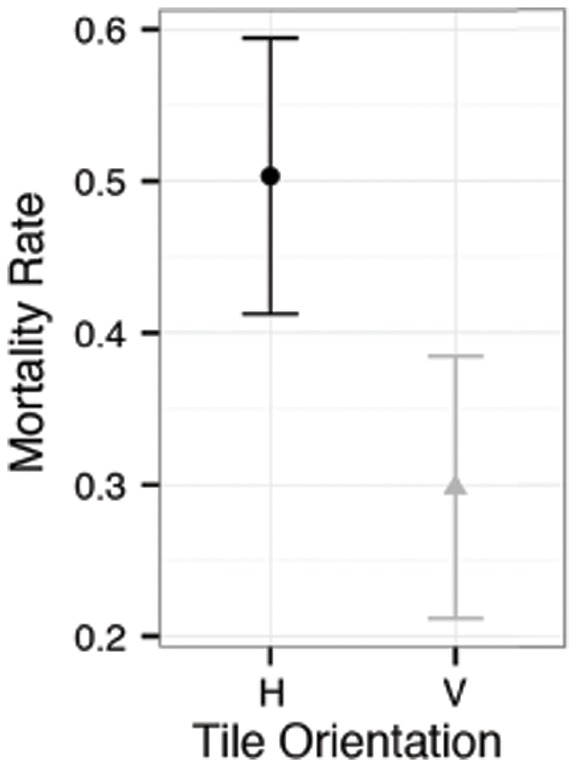
Coral juvenile mortality. Effect of substrata orientation on coral mortality observed between ten and fourteen months. Tile texture and herbivore treatment are not shown, as their effects were not significant. Symbols are means, the whiskers denote 95% confidence intervals. H indicates horizontal tiles and V indicates vertical tiles.

Coral mortality between ten and fourteen months did not differ across herbivore treatment or tile texture, but did differ across tile orientations. A total number of 191 corals succumbed during this time period, 54 were in mollusk treatments, 76 were in urchin treatments, and 61 in control treatments resulting in a 42% mortality rate in the mollusk treatment, 55% in the urchin treatment, and 30% in the control treatment (*P_LRT_*  = 0.560). A mortality rate of 31% was observed on rough textured tiles (126 died) compared with a rate of 49% on smooth tiles (65 died), and again, this difference was also not significant (*P_LRT_*  = 0.150). 50% of corals died on horizontal tiles (79 dead), while 36% died on vertical tiles (112 dead), and this difference was significant (*P_LRT_*  = 0.039) with horizontal tiles experiencing higher mortality (Figure 4). A linear regression was also computed between arcsine squareroot-transformed mortality proportions and coral density. This correlation was not significant (adjR^2^  = 0.104, P = 0.068) but the trend indicated that coral mortality decreased as density increased.

### IV. Coral Colony Size

#### A. *Agaricia*

**Figure 5:**
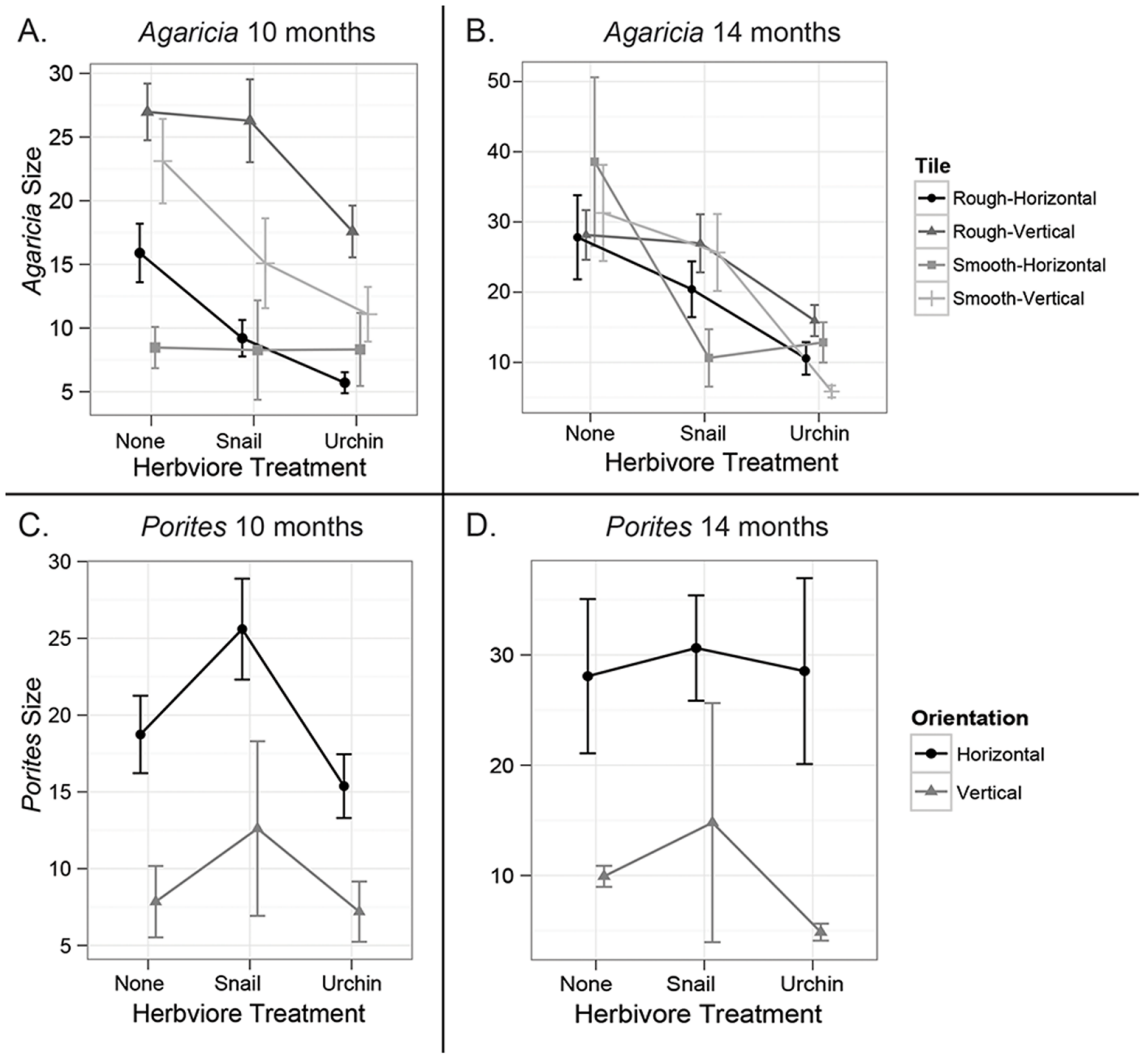
Effects of grazing and substrata interactions on juvenile size through time. I. The effects of herbivore treatment, tile texture and tile orientation on mean *Agaricia* colony size (mm^2^) at ten (A) and fourteen (B) months. II. The effects of herbivore treatment and tile orientation on mean *Porites* colony size (mm^2^) at ten (A) and fourteen (B) months. Tile texture is not shown for *Porites* as its effect was not significant. Symbols are means and the whiskers denote 95% confidence intervals.

After ten months, herbivore treatment (*P_LRT_* <0.001), tile orientation (*P_LRT_* <0.001) and texture (*P_LRT_* <0.024) all had significant effects on mean *Agaricia* colony size (Figure 5A). Tukey's HSD test indicated significant differences between all treatments (Table S1). *Agaricia* colonies in the urchin treatment were the smallest (13.2±1.3 mm^2^) followed by the snail treatment (19.5±2.0 mm^2^) and the largest mean size was observed in the control treatment (23.3±1.6 mm^2^, Figure 5A). *Agaricia* juveniles on vertical tiles were over twice the size (22.3±1.2 mm^2^) of corals on horizontal tiles (9.1±0.8 mm^2^). Colonies were also significantly larger on rough tiles (19.9±1.2 mm^2^) when compared to smooth tiles (15.4±1.6 mm^2^, Figure 5A).

Results for *Agaricia* colony size differed between sampling time points and at fourteen months only herbivore treatment was significant (*P_LRT_* <0.001) and the effects of tile texture and orientation were no longer observed (Figure 5B). A triple interaction was observed between herbivore treatment, tile orientation and tile texture. These interactions were significant (Table S1), however they are difficult to interpret. The main result was that, consistently, corals in the urchin treatment were the smallest, followed by those in the snail treatment while the largest corals were in the control treatment (Figure 5B).

#### B. *Porites*

*Porites* colony size after ten months was significantly affected by substratum orientation (*P_LRT_* <0.001) and herbivore treatment (*P_LRT_* <0.038) while tile texture was not significant (Table 1, Figure 5C). Mean colony size was approximately twice larger on horizontal tiles (20.3±1.7 mm^2^) compared to vertical tiles (10.6±3.4 mm^2^) and urchin grazing reduced colony size (Tukey's HSD, *p*<0.047). Larger coral size on horizontal tiles was opposite to the patterns observed for *Agaricia*, while the reduction in size due to urchin presence was consistent between species.

After fourteen months, substratum orientation continued to have a significant effect (*P_LRT_* <0.001), however the effect of herbivore treatment dissipated through time (Figure 5D). Colony size nearly quadrupled on horizontal tiles (29.2±3.7 mm^2^) when compared to vertical tiles (7.5±2.1 mm^2^), a two-fold increase from observations at ten months (Figure 5D). Interestingly, the number of *Porites* recruits on horizontal tiles decreased while the number of new recruits on vertical tiles doubled in between sampling periods.

### V. *Agaricia* Growth

**Figure 6:**
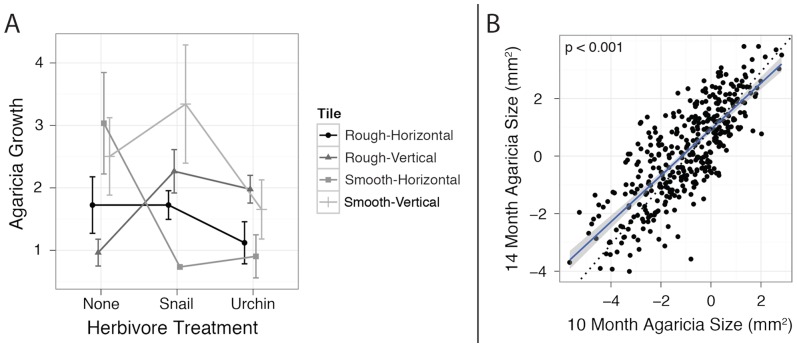
Effects of herbivore treatment, substrata interactions and initial juvenile size on *Agaricia* growth. A. The effect of herbivore treatment, tile texture and tile orientation on *Agaricia* colony growth (mm^2^) over a four month summer period (April-August 2008). B. linear regression of log-transformed *Agaricia* colony size observed in the spring (ten months, horizontal axis) and in the fall (fourteen months, vertical axis). The dotted black line represents the 1∶1 correspondence. The blue line represents the linear regression line and the shaded area represents 95% confidence interval of this regression.

Herbivore treatment had a significant effect on *Agaricia* growth (*P_LRT_* <0.001) and interactions between treatment and texture (*P_LRT_* <0.019) and treatment and orientation (*P_LRT_* <0.017) were also significant (Figure 6A). Colonies in the snail treatment grew an average of 2.12±0.19 mm^2^, colonies in the urchin treatment grew 1.73±0.17 mm^2^ and control colonies grew 1.66±0.22 mm^2^ over the four month summer period. Tukey's HSD tests indicated significant differences between snail and control treatments (p<0.001). A trend towards decreased growth in control relative to urchin treatments was also observed; however this result was not significant (p = 0.058). Many significant interactions were observed, however the major trends suggested that, in the absence of herbivores, *Agaricia* grew less on rough textured tiles, while in the presence of herbivores, *Agaricia* grew more on vertical tiles.

To determine if there was any effect of initial coral size on the coral size four months later, a linear regression was calculated between the log-transformed size after fourteen months and log-transformed size after ten months (Figure 6B). This regression was highly significant (p<0.001) with an adjusted R-squared value of 0.62. Interestingly, the slope of the line was not 1∶1, but rather 0.93 on a log scale indicating that larger corals grew approximately 1.91 fold slower than smaller corals.

To determine if *Agaricia* growth was density dependent, a linear regression was calculated between log-transformed growth and coral density (number of corals per tile). This regression was not significant (adjR^2^  = 0.003, P = 0.313) indicating that *Agaricia* growth in this experiment was independent of coral density on tiles.

## Discussion

Increases in algal biomass and decline of coral recruitment are now observed across many Caribbean reefs (e.g. [44]). Here, we demonstrate that invertebrate herbivory, substratum variations and their interactions significantly affect coral settlement and growth on an Atlantic reef. These effects are species specific, and not always congruent to results observed on other, shallower, Caribbean reefs.

### I. Algal cover

As expected, herbivore treatments were found to significantly reduce algal cover relative to control (Figure S2). This result aligns well with previous literature reporting increases in algae when herbivores were excluded from a system [8], [9], [13]–[15], [22] and, conversely, reduction of algal growth when herbivores are included ([45]–[47]; our study). However, it is important to note that only percent algal cover is quantified here, not actual algal biomass.

### II. Coral Settlement and Post-settlement Mortality

Initially, the goals of this study included assessment of year-to-year variations in coral settlement; however on September 12, 2008 Hurricane Ike passed directly over the FGB with sustained winds of 170 km/h and, despite the 24 m depth of the site and robust construction, destroyed the experimental setup (Figure S1). Therefore, only settlement and survival patterns for the period from August 2007 to August 2008 were documented. This study demonstrated successful coral settlement onto artificial substrata throughout the sampling period (10 and 14 months), a period that allows for sufficient artificial plate fouling [15]. This period included a single mass spawning event by local broadcast-spawning corals (September 2007, personal observation) and covered one brooding coral maximum reproduction season previously reported for the FGB (April-September) [48].

Only two genera of corals recruited in this experiment, *Agaricia* (92.5%) and *Porites* (7.5%) (Figure 2), which are both brooding corals (*Agaricia*: [49]; Caribbean *Porites*: [50]) that constitute minor proportions of natural reef cover at the FGB: 0.08–0.38% (*Agaricia*) and 4.91–8.19% (*Porites*) but occur in high numbers of small individuals [27]. Within these genera, the relative percentages of juveniles roughly matched historical patterns observed on Caribbean reefs. For example, Bak and Engel [51] found that *Agaricia* was the dominant juvenile detected (48–56%) and *Porites* was the second most frequent, at similar depths, in both Curaçao (17–26 m) and Bonaire (17–37 m). Another study in St. Croix also found high recruitment of these two genera, especially relative to their adult abundances on the reef [52]. In the 1980's at the FGB, Baggett and Bright [48] reported that *Agaricia* (76%) and *Porites* (24%) were the only juveniles observed on settlement substrata. The average juvenile density in our study averaged 8.2 corals per tile, 92.5% of which were *Agaricia* (Figure 3). This density recapitulates densities previously observed (6.7 *Agaricia* per tile) at the FGB [48]. More recent studies have also reported these patterns, where agaricids are the dominant recruit and *Porites* are increasingly common [53]. Results here reiterate the observed shift from long-lived broadcast spawning species to weedy brooding species across the Caribbean [5], [54]. Caribbean recruitment studies over the past several decades have consistently found nearly exclusive recruitment of brooding corals ([14], [15], [52] but see [55]).

Annual monitoring of the FGB has shown that coral cover at the east bank is consistently high (50–64%) and reef cover is dominated by two broadcast spawning corals, *Montastraea annularis* species complex (27–34% of reef) and *Diploria strigosa* (6–12% of reef), and suitable coral settlement substrate (crustose coralline algae, fine turfs, and bare rock: 12–24% of reef) [27]. Despite the fact that FGB is one of the few Atlantic reefs still dominated by broadcast spawning species [26], [27], all of which spawned in the summer of 2007 (personal observation), no recruits of these dominant species were found, similar to previous observations [48], [52]. This result may be due to lack of settlement but also, since our first data collection time point was ten months after spawning, may be due to post-settlement mortality, which is known to be very high in broadcast spawning corals (up to 99%) [28], [56]. It is also possible that only some years, perhaps as infrequently as once per decade or less, would enjoy appropriate sea states, currents etc. for sufficient broadcast-spawning coral recruitment to sustain populations [55], [57]. Given the reported lack of recruitment of many key broadcast-spawning coral species across the Caribbean for the past 30 years, it is possible that our results reflect the general local trend.

Although no significant effect of herbivory on the number of coral recruits was observed at ten months, after fourteen months urchin presence was associated with significantly more corals (Figure 3). This result aligns well with previously published data, and is most likely due to algae removal by grazing. For example, a field study in Bonaire observed the highest coral recruitment (73% higher than other treatments) on plates that had low algal biomass, which was the result of herbivore grazing [15]. In natural experiments, first in Jamaica [36], then expanded to sites across the Caribbean [16], it was shown that *D. antillarum* population recovery was correlated with increased coral recruitment, presumable due to grazing.

Two to three times more corals were observed on textured tiles in comparison to smooth tiles (Figure 3), which could be a result of initial settlement preference as well as differential post-settlement survival. Choice during settlement is more likely, since it has been previously shown that coral larvae exhibit strong preferences towards grooves in tiles [38], [40], [58]. This preference might have evolved as a defense from predation or grazing to increase survival [37], [39].

Previous research has described both *Agaricia* and *Porites* recruiting on vertical surfaces of dead coral, demonstrating that recruits prefer cryptic, vertical substrata [59] and, in general, higher coral settlement rates are documented on vertical surfaces [10], [48]. Here total coral numbers were also higher on vertical substrata at both sampling periods (Figure 3) and overall coral mortality was greater on horizontal tiles, indicating variation in post-settlement mortality due to substrata orientation (Figure 4). Since mortality was greater on horizontal surfaces, we are unable to disentangle whether corals are exhibiting settlement bias or our results are due to post-settlement mortality. Lower survival on horizontal surfaces may be due to sedimentation, which has been shown to negatively influence coral settlement, especially in the presence of turf algae [60].

When the number of juveniles per tile are split by genus, at ten months trends were opposite for *Porites* and *Agaricia* (Figure 3). *Agaricia* were three-fold more abundant on vertical tiles, while *Porites* recruits were four times more abundant on horizontal tiles. This striking difference across genera might be due to differences in larval phototactic behavior and/or differences in low-light tolerance [10], and may reflect niche differentiation between these two corals. These results might also suggest variations in post-settlement mortality across genera and perhaps *Agaricia* are more tolerant of low light and less tolerant of sedimentation than *Porites.* Since light is attenuated as depth increases, specific corals may require more exposure to obtain sufficient light for photosynthesis, thereby increasing survival on horizontal surfaces [41]. However, at 14 months, *Porites* were more abundant on vertical tiles. It can be hypothesized that *Porites* corals that settle during the winter months require more light over winter when water turbidity is greater and light cycles are shorter, so they prefer to settle on horizontal tiles. However, this settlement preference might shift to more cryptic habitats during summer months, when more light is available. For the FGB, this hypothesis might be particularly relevant, since it is a relatively deep coral reef (experiment conducted at 24 m). Unfortunately, given our experimental design we are unable to separate whether these results are due to variation in settlement choice or post-settlement mortality.

### III. Coral Size

#### A. *Agaricia*

Urchin grazing has previously been correlated with increased coral recruitment and growth [16], [36], [61], and most herbivore exclusion experiments have shown that when algae are released from grazing pressure, algal growth increases and coral settlement is reduced [8], [9], [13], [14], [60]. Contrary to this expectation, we found that *Agaricia* colony size was in fact significantly reduced in herbivore treatments (Figure 5A, B). *D. antillarum* presence reduced *Agaricia* size almost two-fold, from 23.3±1.6 mm^2^ in no-herbivore controls down to 13.2±1.3 mm^2^ at the 10-month sampling period, and this size reduction was still evident at 14 months (Figure 5A,B). One explanation for this size reduction could be destructive overgrazing by urchins, which might have occurred since natural *D. antillarum* densities are currently low at the FGB (0.05 individuals/m^2^) [27], much lower than pre-1984 levels, which ranged from 0.54–1.63 individuals/m^2^ between 1970 and 1983 [62], [63]. Destructive overgrazing occurs when echinoid densities are high enough or algal cover is low enough that grazing becomes competitive and abrasive, causing reduced coral growth and accidental coral consumption [14], [64]. Korzen *et* *al.,* [46] also observed a negative effect of urchins on coral survival; however most studies find larger spat in grazed treatments (e.g. [15]). Since FGB is an uncommonly deep reef (24 m at the field site), algae at this site presumably grows slower than at other, shallower, Caribbean reefs, and this coupled with the naturally low urchin densities on the reef may have resulted in more intensive grazing by herbivores in the experimental treatment. Alternatively, the reduced average size of recruits in presence of herbivores might be due to higher continuous recruitment, leading to enrichment of the recruit population by the younger, smaller corals. Given that there was a significant increase in *Agaricia* recruitment to urchin grazed tiles at 14 months, this explanation appears equally likely.

Tile texture and orientation both had strong effects on *Agaricia* size: the recruits were significantly larger on rough and vertically oriented tiles at ten months (Figure 5A, B). Interestingly though, size differences observed at ten months were no longer detectable at fourteen months. This effect loss over time may be due to seasonality and/or due to the settlement tiles becoming completely encrusted by this stage.

#### B. *Porites*

In contrast to *Agaricia*, *Porites* colonies on horizontal tiles were four times the size of those on vertical substrata suggesting that *Porites* not only prefers to settle in exposed habitats, as discussed in the previous section, but also grow better there, perhaps due to better sunlight sequestration [51]. This result is additional data suggesting niche partitioning between these two coral genera. Similarly to *Agaricia,* at ten months, *Porites* recruit size was also reduced in urchin treatments, however at fourteen months, *Porites* colonies were not significantly affected by tile texture or herbivore presence (Figure 5C, D). Nevertheless, it is important to note that this lack of significance may be due to the lower recruit numbers of this species making subtle differences in coral size difficult to ascertain statistically.

### IV. *Agaricia* Growth

Herbivory has been shown to enhance coral growth [7], [22], and here we detected the same trend by tracing the size of individual *Agaricia* recruits over four summer months (Figure 6A). As expected, increased growth in herbivore treatments was observed most likely due to algae being grazed thereby reducing space competition with corals [3]. Interesting interactions for *Agaricia* growth were also observed with higher growth on rough textured tiles in the presence of herbivores and on vertical tiles when herbivores were not present. Previous studies have found that corals prefer to settle in cryptic habitats to avoid predation or accidental overgrazing [38]–[40] and this is likely the case in this study since no variation in survival due to texture was observed, indicating that *Agaricia* do indeed grow faster on textured tiles. Recruits of all sizes exhibited similar proportional increases in size (Figure 6B), however, the slope of the regression was significantly less than one suggesting that larger corals grow approximately 1.91 fold slower than smaller corals. This observation has previously been shown for other cnidarians, where size-dependent growth rates were observed [65]. Size-dependent growth is not always the case for all species [65], [66], however for *Agaricia* juveniles, it appears that smaller corals grow faster.

### V. Conclusions

Previous research has demonstrated that grazing is critical to reef resilience and herbivores play a primary role in creation of open space for corals to settle [7], [61]. The present study further highlights the importance of herbivory on reefs, demonstrating that herbivores effectively remove algae from the substrata allowing space for corals to settle increasing overall recruitment. However, this study also suggests that although the presence of herbivores leads to less algal cover and a moderate increase in coral settlement, this presence might have a negative effect on coral size. The effects of herbivory also depends on substratum orientation and type, as well as on coral species, highlighting the ecological complexity and niche partitioning in coral reef communities.

## Supporting Information

Figure S1 **Experimental timeline.** Timeline highlighting platform construction, experimental changes due to weather and data collection time points.
(TIF)

Figure S2 **Effects of herbivores on algae cover.** A. Percent algal cover at three months after tiles deployment (September 2007) in three different herbivore treatments. Asterisks indicate significant differences as determined by Tukey's HSD tests obtained using arcsine-square root transformed data. B. The sea urchin *Diadema antillarum.* C. The snail *Cerithium litteratum*.
(TIF)

Table S1 **Tukey**'**s HSD statistics for each experiment (algae cover, number of coral juveniles, coral size, coral mortality and** ***Agaricia*** **growth) in response to herbivore treatment and tile texture and orientation.** Only significant terms are included in the table.
(DOCX)
