# Supplementary figures and images for: Ecological Complexity of Coral Recruitment Processes: Effects of Invertebrate Herbivores on Coral Recruitment and Growth Depends Upon Substratum Properties and Coral Species
Source: PLoS One. 2013 Sep 9;8(9):e72830. doi: 10.1371/journal.pone.0072830 (PMC3767691; doi:10.1371/journal.pone.0072830)

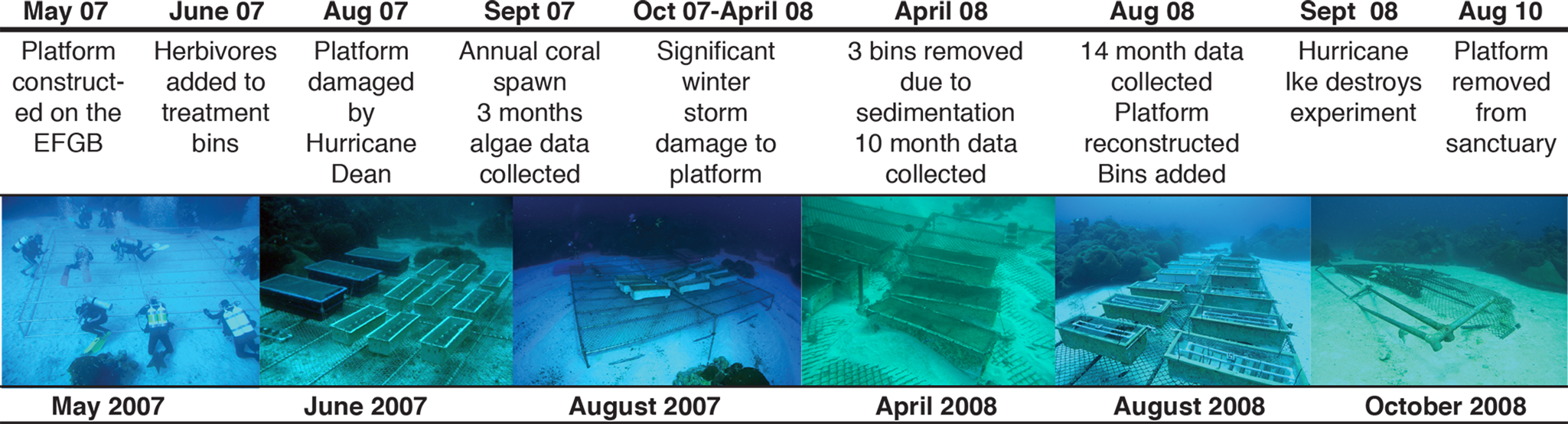

Supplement: Figure S1 — Experimental timeline. Timeline highlighting platform construction, experimental changes due to weather and data collection time points. (TIF) [file pone.0072830.s001.tif]

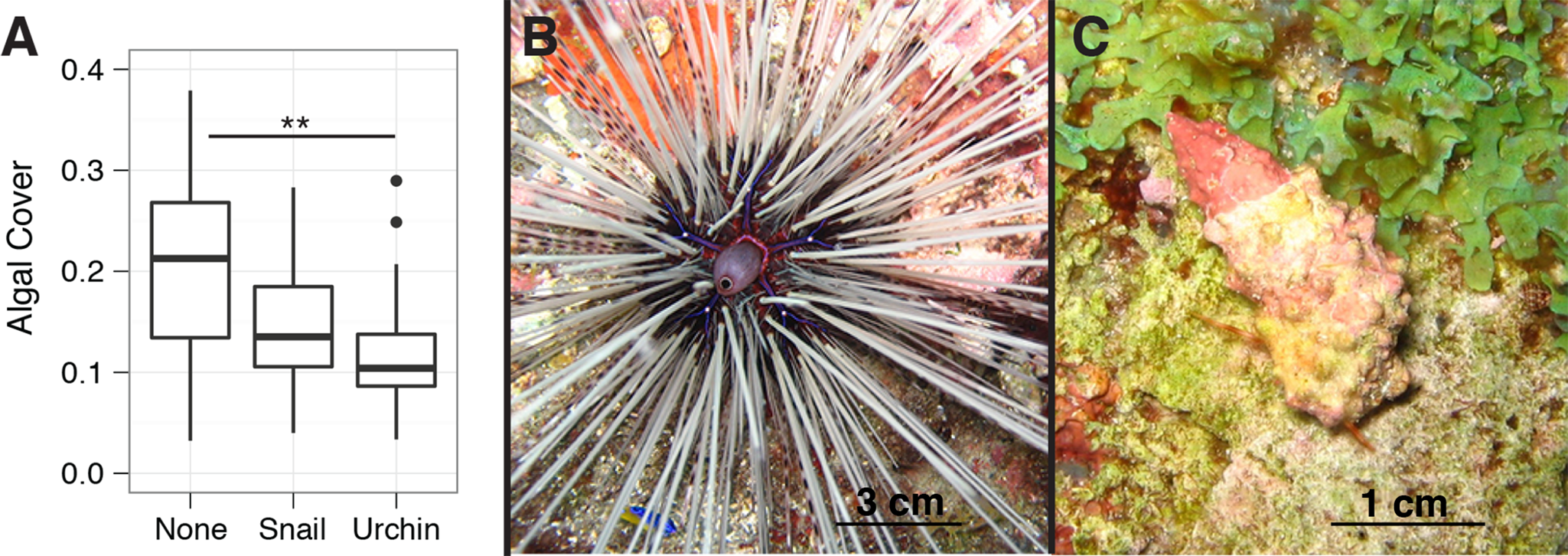

Supplement: Figure S2 — Effects of herbivores on algae cover. A. Percent algal cover at three months after tiles deployment (September 2007) in three different herbivore treatments. Asterisks indicate significant differences as determined by Tukey's HSD tests obtained using arcsine-square root transformed data. B. The sea urchin Diadema antillarum. C. The snail Cerithium litteratum. (TIF) [file pone.0072830.s002.tif]
