# Supplementary material for: Ecological Complexity of Coral Recruitment Processes: Effects of Invertebrate Herbivores on Coral Recruitment and Growth Depends Upon Substratum Properties and Coral Species
Source: PLoS One. 2013 Sep 9;8(9):e72830. doi: 10.1371/journal.pone.0072830 (PMC3767691; doi:10.1371/journal.pone.0072830)
Supplement: Table S1 — Tukey's HSD statistics for each experiment (algae cover, number of coral juveniles, coral size, coral mortality and Agaricia growth) in response to herbivore treatment and tile texture and orientation. Only significant terms are included in the table. (DOCX) [file pone.0072830.s003.docx]

Table S1

| **Experiment** | **Factor** | ***p*** |
| --- | --- | --- |
| Algae Cover | Treatment |  |
| (3 months) | U - C | 0.002 |
| Coral Numbers | Oreintation*Texture |  |
| (10 months) | S:H – R:H | 0.002 |
|  | R:V – R:H | <0.001 |
|  | R:V – S:H | <0.001 |
|  | S:V – R:V | <0.001 |
| Coral Numbers | Treatment |  |
| (14 months) | U - C | <0.001 |
|  | U - M | <0.001 |
|  | Orientation*Texture |  |
|  | V:R - H:R | <0.001 |
|  | H:S – H:R | 0.046 |
|  | H:S –V:R | <0.001 |
|  | V:S –V:R | <0.001 |
|  | V:S –H:S | <0.001 |
|  | Orientation*Treatment |  |
|  | V:C – H:C | <0.001 |
|  | V:M – H:C | <0.001 |
|  | V:U – H:C | <0.001 |
|  | H:M – V:C | 0.013 |
|  | V:U – V:C | <0.001 |
|  | V:M –H:M | <0.001 |
|  | V:U – H:M | <0.001 |
|  | H:U – V:M | 0.003 |
|  | V:U – V:M | <0.001 |
|  | V:U – H:U | <0.001 |
|  | Texture*Treatment |  |
|  | R:U – R:C | <0.001 |
|  | R:M – S:C | <0.001 |
|  | R:U – S:C | <0.001 |
|  | S:M – R:M | <0.001 |
|  | R:U – R:M | 0.013 |
|  | R:U – S:M | <0.001 |
|  | S:U – R:U | <0.001 |
|  | Orientation*Texture*Treatment |  |
|  | V:R:C – H:R:C | 0.043 |
|  | V:R:M –H:R:C | <0.001 |
|  | V:R:U –H:R:C | <0.001 |
|  | H:S:C – V:R:C | <0.001 |
|  | H:S:M – V:R:C | 0.001 |
|  | V:R:U – V:R:C | <0.001 |
|  | H:S:U – V:R:C | 0.029 |
|  | V:R:M – H:S:C | <0.001 |
|  | V:R:U – H:S:C | <0.001 |
|  | V:S:U – H:S:C | <0.001 |
|  | V:R:M – V:S:C | 0.001 |
|  | V:R:U – V:S:C | <0.001 |
|  | V:R:M – H:R:M | <0.001 |
|  | V:R:U – H:R:M | <0.001 |
|  | H:S:M – V:R:M | <0.001 |
|  | V:S:M – V:R:M | <0.001 |
|  | H:R:U – V:R:M | <0.001 |
|  | V:R:U – V:R:M | <0.001 |
|  | H:S:U – V:R:M | <0.001 |
|  | V:R:U – H:S:M | <0.001 |
|  | V:S:U – H:S:M | 0.003 |
|  | V:R:U – V:S:M | <0.001 |
|  | V:R:U – H:R:U | <0.001 |
|  | H:S:U – V:R:U | <0.001 |
|  | V:S:U – V:R:U | <0.001 |
|  | V:S:U – H:S:U | 0.049 |
| *Agaricia* Colony | Treatment |  |
| Size (10 months) | M – C | 0.002 |
|  | U – C | <0.001 |
|  | U – M | 0.012 |
| *Agaricia* Colony | Treatment |  |
| Size (14 months) | M – C | 0.003 |
|  | U – C | <0.001 |
|  | U – M | <0.001 |
|  | Orientation*Texture*Treatment |  |
|  | V:S:U – H:R:C | 0.001 |
|  | H:S:M – V:R:C | 0.046 |
|  | H:R:U – V:R:C | 0.032 |
|  | V:R:U – V:R:C | <0.001 |
|  | V:S:U – V:R:C | <0.001 |
|  | V:R:U – H:S:C | 0.045 |
|  | V:S:U – H:S:C | 0.003 |
|  | V:R:U – V:S:C | 0.017 |
|  | V:S:U – V:S:C | <0.001 |
|  | V:S:U – H:R:M | <0.001 |
|  | V:S:U – V:R:M | 0.001 |
|  | V:S:U – V:S:M | 0.001 |
| *Porites* Colony | Treatment |  |
| Size (10 months) | U - M | 0.047 |
| *Agaricia* Colony | Treatment |  |
| Growth | M – C | <0.001 |
|  | U – C | 0.058 |
|  | Texture*Treatment |  |
|  | S:C – R:C | <0.001 |
|  | R:M – R:C | <0.001 |
|  | R:U – R:C | 0.002 |
|  | Treatment*Orientation |  |
|  | V:M – V:C | <0.001 |
|  | V:M – H:U | 0.045 |
|  | V:U –V:C | 0.006 |

Herbivore Treatments: M=snail (mollusk), U=urchin, C=no herbivore control

Tile textures: S=smooth, R=rough

Tile orientations: H=horizontal, V=vertical
